# Changes in Disability, Physical/Mental Health States and Quality of Life during an 8-Week Multimodal Physiotherapy Programme in Patients with Chronic Non-Specific Neck Pain: A Prospective Cohort Study

**DOI:** 10.1371/journal.pone.0118395

**Published:** 2015-02-24

**Authors:** Antonio Ignacio Cuesta-Vargas, Manuel González-Sánchez

**Affiliations:** 1 School of Clinical Sciences, Faculty of Health at the Queensland University of Technology, Brisbane, Australia; 2 Departamento de Psiquiatría y Fisioterapia, Facultad de Ciencias de la Salud, Universidad de Málaga, Andalucia Tech, Cátedra de Fisioterapia y Discapacidad, Instituto de Biomedicina de Málaga (IBIMA), Grupo de Clinimetria (FE-14), Malaga, Spain; Copenhagen University Hospital, Hvidovre, DENMARK

## Abstract

**Aim:**

The aim of this study was to analyse the effect of an 8-week multimodal physiotherapy programme (MPP), integrating physical land-based therapeutic exercise (TE), adapted swimming and health education, as a treatment for patients with chronic non-specific neck pain (CNSNP), on disability, general health/mental states and quality of life.

**Methods:**

175 CNSNP patients from a community-based centre were recruited to participate in this prospective study. Intervention: 60-minute session (30 minutes of land-based exercise dedicated to improving mobility, motor control, resistance and strengthening of the neck muscles, and 30 minutes of adapted swimming with aerobic exercise keeping a neutral neck position using a snorkel). Health education was provided using a decalogue on CNSNP and constant repetition of brief advice by the physiotherapist during the supervision of the exercises in each session. Study outcomes: primary: disability (Neck Disability Index); secondary: physical and mental health states and quality of life of patients (SF-12 and EuroQoL-5D respectively). Differences between baseline data and that at the 8-week follow-up were calculated for all outcome variables.

**Results:**

Disability showed a significant improvement of 24.6% from a mean (SD) of 28.2 (13.08) at baseline to 16.88 (11.62) at the end of the 8-week intervention. All secondary outcome variables were observed to show significant, clinically relevant improvements with increase ranges between 13.0% and 16.3% from a mean of 0.70 (0.2) at baseline to 0.83 (0.2), for EuroQoL-5D, and from a mean of 40.6 (12.7) at baseline to 56.9 (9.5), for mental health state, at the end of the 8-week intervention.

**Conclusion:**

After 8 weeks of a MPP that integrated land-based physical TE, health education and adapted swimming, clinically-relevant and statistically-significant improvements were observed for disability, physical and mental health states and quality of life in patients who suffer CNSNP. The clinical efficacy requires verification using a randomised controlled study design.

**Trial Registration:**

ClinicalTrials.gov NCT02046876

## Introduction

Chronic neck pain (CNP) is defined as pain or intense discomfort in the lateral area or back of the neck with an establishment and/or persistence period of over 12 weeks [1]. When the aetiology of cervical pain is unknown, neck pain is defined as non-specific [1]. This is a very common musculoskeletal problem in developed countries [2]. The prevalence of CNP in a year varies between 17% and 75% [1–3]. Also, the rates of recurrence and chronicity are over 35% for subjects who suffer neck pain [2,4–6].

The socio-economic system is seriously affected by chronic non-specific neck pain (CNSNP) due to direct costs affecting the health system (25% of visits to chiropractors, 15% to hospital physiotherapists, 2% to family physicians as well as 75% of musculoskeletal assessments by rheumatologists are related to neck pain [7] and indirect costs resulting from absences from work, lower productivity and even early retirement [1,8]. Due to the socio-economic burden of neck pain, it is important to maximise effective strategies to improve function, limit the progression of degenerative changes and prevent future neck pain relapses [8,9].

Therapeutic exercise (TE) is a common treatment for CNP sufferers [2,9–11]. Physical exercise is used to improve physical function and reduce the symptoms of pain and stiffness [1,2,7,8,10]. Intervention in water has been established as a therapeutic modality that uses the physical properties of water to produce physical and functional improvements in patients [12]. It has been demonstrated that TE in water is effective in improving functional capacity and symptoms in patients with CNP [1,2,9,13,14]. Both health education and posture are important components of the overall treatment of neck pain [8]. Brief advice, verbal instruction and reinforcement through guidance leaflet sheets enable a reconceptualisation of the problem by the patient [8]. It has been demonstrated that not only personal fitness but also psychological and emotional states may influence pain perception [8], and these could be integrated during a TE programme [14–19]. Furthermore, functional cognitive therapy has been proven as effective in overcoming the fear of movement displayed by chronic musculoskeletal disease sufferers by reshaping their attitudes and beliefs about their pain [20,21]. Specifically in respect to therapeutic physical exercise in water, some modalities such as adapted swimming are presented as a feasible clinical practice for patients with CNSNP, eliminating fear of movement as it is an accessible and fun activity [22]. Interventions that integrate physical activity, adapted swimming and health education for CNSNP sufferers have not been discussed in the literature to date.

The aim of this study was to analyse the effect of an 8-week multimodal physiotherapy programme (MPP), integrating physical land-based TE, adapted swimming and health education, as a treatment for patients with CNSNP, on disability, general health/mental states and quality of life. The hypothesis was that the MPP would improve disability, general health/mental states and quality of life in patients suffering CNSNP.

## Methods

### Design

The prospective study analysed the effect of a MPP, integrating physical land-based TE, adapted swimming and health education, as a treatment in patients with CNNSP for 8 weeks. The protocol for this trial and supporting TREND checklist are available as supporting information; see S1 TREND Checklist and S1 Protocol.

### Participants

A consecutive sample of 175 CNSNP volunteers from a community-based centre (Patronato de Deportes de Torremolinos—Málaga (Spain)) were recruited to participate in this prospective study.

Inclusion and exclusion criteria

The inclusion criteria used were: to be between 18 and 65 years old, suffering CNSNP (persistent neck pain for more than 12 weeks) and delivery of informed consent. The exclusion criteria used were: neck pain due to an identifiable cause (herniated disc, spinal fracture, metastatic cancer, severe or progressive scoliosis, infection, spinal stenosis, unexplained weight loss, spondylolisthesis, osteoporosis, spinal stenosis, inflammatory rheumatic disease); surgery on the spine in the past year; traffic accident in the last 3 months; cervical radiculopathy; and pregnancy. Also excluded were patients who suffered a condition that could alter the effects of treatment (untreated depression, medical treatment for neck pain, heart or lung disease, severe fibromyalgia and medication used for AIDS or hepatitis) and those who could not swim and/or presented treatment difficulty (dementia, serious addiction problems, vision deficit or paralysis).

### Ethics statement

All subjects provided written informed consent. This study was conducted in accordance with the Ethical Principles for Medical Research Involving Human Subjects [23]) and approved by the Ethics Committee of Malaga University. All the laws of Spain on data protection (LO 15/1999 and Royal Decree 1720/2007) were respected to guarantee the anonymity of the patients at all times and for the storage of their personal data. The data were stored on a computer encrypted and protected by an encryption code. Only researchers in this study had access to the data.

### Study outcomes

The primary outcome variable of this study was the disability of patients suffering CNSNP. To measure the effect of the MPP on these patients, the Spanish version of the Neck Disability Index (NDI) was used [2,24], which has a reliability of (intraclass correlation coefficient, ICCs = 0.98 [25]. The primary outcome variable (NDI), was selected to track the changes in the patient’s disability, as a result of CNP, before and after the MPP. The absolute range value of the primary variable is from 50 (maximum disability) to 0 (no disability). The secondary outcome variables were physical and mental health states and quality of life of patients. To measure changes in these variables, the Spanish version of the SF-12 (scale 0–100) and EuroQoL-5D (scales: 0–1 (quality of life) and 0–100 (quality of life visual analogue scale or VAS)) questionnaires were used. The reliability of both instruments, expressed in ICCs, are 0.86 and 0.89 (SF-12) [26] and 0.86 and 0.90 (EuroQoL-5D) [27] respectively. All measurements were taken at baseline and after the intervention (8 weeks) by an investigator blinded and external to the study.

### Intervention

The MPP, in which land-based physical TE, adapted swimming and health education were integrated as a treatment for CNSNP suffers, was performed three times a week for 8 weeks.

Each 60 minute session consisted of 30 minutes of land-based exercise dedicated to improving mobility, motor control, resistance and strengthening of the neck muscles [1,2,14], and 30 minutes of adapted swimming with aerobic exercise keeping a neutral neck position through use of a snorkel [28]. A scheme of the exercises, muscles on which they intervene and intensity of the exercises can be observed in Table 1. A blinded physiotherapist supervised the session including the exercise workloads and educational behaviour intervention [14,18,19]. Self-stretching of myotendinous barriers of pectoralis, levator scapulae, upper trapezius and scalenus was used to restore mobility [1,2]. Motor control was improved by activating the local system for 10 seconds in a neutral spine position (checking the whole spine from pelvis to head) by trial and error; participants were actually doing this in their daily lives outside of the therapy sessions (such as when sitting, walking and using a computer). Manual teaching and mirrors were used for feedback [29–31]. The isometric muscular endurance of deep neck muscles was improved by synergistic arm exercises, such as a press or pullover using dumbbells. Exercises were applied in three sets of 15 repetitions (30 seconds of exercise with 30 seconds of rest), while keeping a cervical neutral position. Rubber band exercises were used with the same criteria, as they also activate scapular fixators, including push and pull exercises.

**Table 1:** Scheme of the performed land-based and adapted swimming exercises and their intensity.

	Type of Exercises	Muscles	Name of Exercise	Intensity
Land-Based Exercise (30 minutes)	Stretching (Mobility)	Pectoralis Major Muscle	Maximum retroflexion with external rotation and horizontal abduction of the shoulder. Both sides simultaneously.	3 × 30 seconds
		Scapula Muscles	Maximum external rotation with shoulder abduction, allowing an external bell scapula lowering the medial angle of it. Contralateral cervical rotation and flexion of the head.	
		Trapezius Muscle (2 Exercises)	Head forward, with the hands behind the head and pushing slightly downward. Pass the ipsilateral hand behind the waist. Head flexion with contralateral rotation and tilt. Support hand on head and drop weight without straining.	
	Resistance Strengthening	Pectoralis Major Muscle Shoulder Stabiliser Muscles	Side Lateral RaiseBarbell Bench Press	3 × 15–20 reps 20 second rest 50%–60% 1RM
		Internal Rotator Muscles Shoulder External Rotator Muscles	Cable Internal Cuff Rotation Cable External Cuff Rotation	
		Trapezius Muscle Latissimus Dorsi Muscle Serratus Muscle	Lat Pull Down Straight Arm Pull Down	
	Motor Control	Subjects should complete all repetitions of the series at the indicated intensities without altering the execution position and without losing cervical lordosis.		
Adapted Swimming (30 minutes)	Front Crawl Swimming	Using a Snorkel		Subjectively comfortable intensity for the patient.
	Front Crawl Swimming (Underwater Recovery)			

The session also consisted of 30 minutes of adapted swimming with aerobic exercise performed in water keeping a neutral neck position. A snorkel was used to enable aerobic exercise without overloading neck structures. Ventral swimming on the front with a snorkel reduces head weight because of buoyancy and avoids any neck movement for breathing [28]. The main factor that affects the neck is the propulsive force from the upper limbs. By controlling speed and the range of motion of limbs, the therapist can set an exercise where the neck is in a neutral position and deep muscles are stimulated, in addition to the aerobic exercise benefits. Health education was provided using a decalogue on CNSNP and constant repetition of brief advice by the physiotherapist during the supervision of the exercises in each session [2].

All participants were encouraged to take an active role in the MPP. Furthermore, the importance of adherence to treatment was emphasised. Patients were grouped into sessions of 8–10 patients and sessions were conducted and continuously supervised by a physiotherapist. The participants were introduced into existing intervention groups in the community-based centre. The physiotherapist responsible for the supervision of each group did not know which patients were part of the present study.

Recruitment of participants and data collection took place between January and June 2013. Both the study protocol and the measured patients are unique to this study. For these reasons, the present study was registered with the following clinical trial reference: NCT02046876.

### Statistical analysis

Data were obtained at baseline and after the intervention (8 weeks). Prior to any analysis, a study of normal outcomes using the Kolmogorov–Smirnov test was performed. Descriptive analyses were conducted (including measurements of central tendency and dispersion, which were calculated for all outcome measurements). Subsequently, differences between baseline data and that at the 8-week follow-up were calculated for all outcome variables using the paired Student’s t-test (the distribution of the sample for all variables was parametric). The analyses were performed as per-protocol. Data analyses was performed using the Statistical Package for the Social Sciences (version 17.0 for Windows, Illinois, USA).

### Sample size

A priori sample size calculation indicated 81 patients were required to detect a minimum important difference of 10.5 points in NDI score [32] (effect size d = 0.28, alpha = 0.05, beta = 0.08)[33] using G*Power 3.1 software. As a pragmatic approach, we decided to included as many patients as possible during the pre-planned recruitment period, which is why the final sample ended up being 175 patients.

## Results

**Fig 1:**
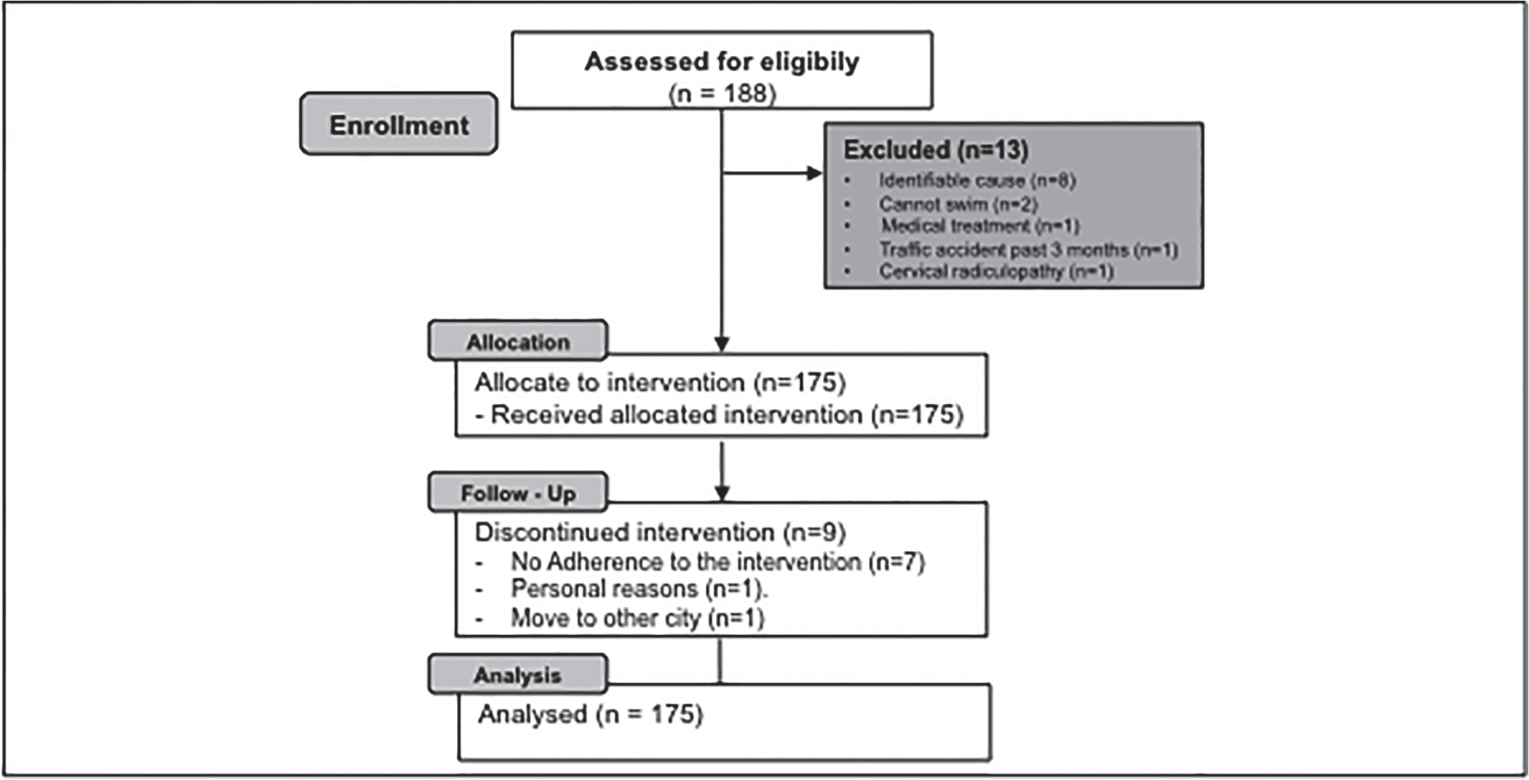
Flowchart of patient enrollment.

A sample of 175 CNSNP sufferers was selected to begin the study. Nine patients were not included in the data analysis: seven patients did not achieve the necessary adherence to the programme; one patient left the treatment for personal reasons; and one patient moved to another city. Of the remaining 166 subjects, 53% were women. Fig. 1 shows a diagram of the recruitment, intervention, follow-up and data analysis processes of the study. In addition, the descriptive data for the sample can be observed in Table 2. It includes the outcome variables (primary and secondary) at the beginning of the study and a confidence interval at 95%.

**Table 2:** Characteristics of the study participants at baseline.

	MEAN	CONFIDENCE INTERVAL (95%)	
		Min	Max
**Age** (years)	45.6	31	59
**Size** (cm)	164.5	140	189
**Weight** (Kg)	97.6	45.5	189.0
**Body Mass Index** (kg/m^2^)	25.9	19.3	33.4
**NDI** (50–0)	28.2	19	33
**SF12 Ph 1** (0–100)	39.9	12.6	59.9
**SF12 Mt 1** (0–100)	40.6	14.3	67.9
**EuroQoL 5D 1** (0–1)	0.7	-0.6	1.0
**EuroQoL VAS 1** (0–100)	55.5	4	100
N	**175**		

**NDI**: Neck Disability Index **SF12 Ph**: Short form 12 physical health state. **SF12** Mt: Short form 12 physical mental state.

Table 3 presents the mean values of each outcome variable before and after the intervention and the mean difference between the two measures. In the present study, a significant improvement of 11.32 (± 8.42) points in disability (NDI) in patients who suffer CNSNP after 8 weeks of intervention was observed. This improvement was an absolute value on a scale of 50 to 0, so the average percentage improvement was 22.64%. The observed changes in physical and mental health states show significant improvements of 14.36 (physical) and 16.29 (mental). In terms of quality of life, patients experienced a significant improvement of 0.13 (13%) and 15.16% using the EuroQoL-5D and EuroQoL-VAS respectively.

**Table 3:** Mean differences between the outcomes variables pre- and post-intervention.

	Baseline			Post-intervention			Difference		
	MEAN	CI95%		MEAN	CI95%		MEAN (%)	CI95%	p-value
**Disability** NDI (50–0)	**28.20**	19.0	33.1	**16.88**	10	23.3	-**11.32** (22.64%)	7.7 to 14.8	**<0.001**
**Physical Health State** (SF12—Physical) (0–100)	**39.96**	12.6	59.9	**54.32**	22.5	61.6	**14.36** (14.36%)	12.5 to16.7	**<0.001**
**Mental Health State** (SF12—Mentall) (0–100)	**40.63**	14.3	67.9	**56.92**	23.4	66.9	**16.29** (16.29%)	14.1 to 18.8	**<0.001**
**Quality of Life (5 dim)** EuroQoL 5D (0–1)	**0.70**	-0.6	1.0	**0.83**	0.04	1.0	**0.13** (13.00%)	0.04 to 0.19	**<0.001**
**Quality of Life VAS** EuroQoL VAS (0–100)	**55.59**	4	100	**70.74**	14	97	**15.16** (15.16%)	13.5 to 16.7	**<0.001**
**N**	**175**								

CI95% = 95%Confidence interval.

## Discussion

This study aimed to analyse the medium-term effect of a MPP, which integrated physical land-based TE, adapted swimming and health education as a treatment (8 weeks), on disability, general health/mental states and quality of life for patients who suffer CNSNP. Based on the results, where significant improvements in disability, quality of life and physical and mental health states were observed, it is possible to affirm that the aim of this study was achieved and the hypothesis was confirmed.

All variables analysed were self-reported by the patients, thus the patient’s perception of the impact of the intervention on the CNSNP was assessed.

### Change in disability

Comparing the changes in disability with those produced in regard to physical and/or mental health state, virtually all studies show how disability is the variable that shows the best response to the presented treatment. The relative average improvement in disability (NDI) in patients who suffer CNSNP after 8 weeks of intervention with the MPP was 22.64% (11.32 (± 8.42) points on NDI scale) and these results are partially consistent with previous studies [34–38]. Recent studies which have used exercise as an intervention for patients who suffer CNSNP have shown improvements in disability of 1.5% [35], 8.48% (NDI: 4.24 points) [35], 14% (NDI: 7.0 points) [37], 11% and 19.8% (NDI: 5.5 and 9.9 points respectively) [37] and 19.2% and 28.6% (NDI: 14.3 points) [38]. The difference in results between the present study and those previously reported may have various explanations. First, the degrees of disability that participants from different studies have are important. In the present study, all participants had a moderate disability (NDI: 28.20 (± 13.08) (Table 3)). In some studies, the level of disability of participants was similar that of the present study. For example, participants of the study published by Bronfort et al. [38] had levels of disability between 28.25 (± 6.40) and 28.45 (± 7.1). Similarly, levels of disability among study participants in Michalsen et al.’s research [36] were also moderate (25.4 ± 5.2 to 25.8 ± 5.5). However, in the studies published by Mallin and Murphy (35) and Evans et al. [37], disability levels of the participants were mild, with values ranging from NDI 13.4 (± 4.5) [37] to 14.00 (± 6.35) [35]. These initial levels of disability should force us to carefully analyse the evolution of the participants between the studies as the scope for participant improvement in this study is broader than the scopes for improvement in the studies of Evans et al. [37] and Mallin and Murphy [35].

The level of adherence of participants in each study may also be another explanation for the different results between the present study and the studies analysed. In the study by McLean et al. [34], adherence of the study participants was very low (47%), affecting the results negatively. Moreover, both in the study of Mallin and Murphy [35] and Michalsen et al. [36], the patients received intervention through exercise-specific methodologies. The first one employed Pilates, while the second one used yoga as the intervention method. However, when comparing studies that include TE intervention as the primary method, consistent results between the present study and previously published results were observed [37,38]. One explanation for the small differences observed between the three studies can be found in the type of exercise proposed by each protocol. Evans et al. [37] proposed exercises based on strength endurance while Bronfort et al. [38] proposed stretching and aerobic endurance, while in this study all types of exercises (stretching, strength endurance and aerobic endurance) were integrated. On the other hand, the distribution of sessions may also have influenced the results, since in both studies 20 sessions were distributed over 12 [37] and 11 [38] weeks respectively (on average, less than two sessions weekly). However, in the present study, 24 sessions were distributed over 8 weeks of intervention (three sessions weekly).

### Changes in physical and mental health states

The improved percentages in physical (14.36%) and mental (16.29%) health states are consistent with the study presented by Bronfort et al. [38], where patients experienced an improvement of 12% after 11 weeks of intervention. However, the range of improvement experienced by participants in this study is higher than in the study published by Bronfort et al. [38]. The present study provides baseline values of 39.96 (± 12.63) and 40.63 (±14.35) for general physical and mental health states respectively (Table 3), while the participants in Bronfort et al.’s study [38] had values of 69.0 (± 13.1) on a general health state scale of 0–100 (SF-12 and SF-36). However, consistency in results was not observed in other studies analysed because significant improvements in physical health state of 8% and 0.6% [36] and mental health state of 3.3% [37] and −2.4% [36] respectively were observed, although the initial levels of general physical and mental health were similar between the present study and Michelsen et al.’s [36] study (physical: 38.5 (± 7.1) to 40.7 (± 6.0) and mental: 44.3 (± 11.7) to 43.0 (± 10.4)).

Moreover, for the reasons outlined above in regard to the type of exercise proposed and distribution of the sessions throughout the intervention, another possible explanation for these different results could be found in the use of health education as a tool to integrate the activities learnt during treatment sessions into the daily life of each patient [8]. This progressive learning that is acquired throughout the intervention is easily transferable to the activities of the daily lives of the patients, positively affecting their physical and mental health states [8].

### Changes in quality of life

The improvements shown by patients in quality of life (13% (0.13 points on EuroQoL-5D scale) and 15.16% (EuroQoL-VAS)) are consistent with a previous study published on patients who suffer CNSNP [14], where the patients experienced an improvement of 10% (EuroQoL-5D: 0.10) and 5.22% (EuroQoL-VAS). It is also important to consider that the baseline values in quality of life of the participants in both studies were similar, with values on EuroQoL-5D and EuroQoL-VAS of 0.70 (± 0.21) and 55.59 (± 19.15) in the present study and 0.70 (± 0.26) and 60.81 (± 22.65) in the study published by Cuesta-Vargas et al. [14] respectively.

In addition, the consistency in the results for quality of life between both studies could be explained by the fact that in both studies the patients performed a MPP where physical land-based TE, adapted swimming and health education were integrated, and patients were chronic musculoskeletal disease sufferers (CNSNP in this study, and CNSNP, non-specific chronic lower-back pain and osteoarthritis patients in the previous study [14].

### Strengths and weaknesses of the study

To our knowledge, this study is the first to integrate physical land-based TE, adapted swimming and health education as a treatment for patients who suffer CNSNP. Furthermore, it was observed that this procedure produced clinically relevant improvements in all the variables examined. However, it also has some limitations such as lack of a control group, which would have strengthened the results. The influence of different anchors for the NDI in minimal important change would be considered for subgroups of patientswith higher or lower baseline scores [39]. The effect was only measured in the medium term (8 weeks); the analysis of the treatment effect could be extended to a longer term. Finally, this procedure was performed with patients with CNSNP, thus this intervention protocol is limited to such patients, and we should be cautious about its application in respect to other types of patients.

Study participants benefitted from clinically significant improvements from the joint action of three methods of intervention (TE, health education and adapted swimming) which have proven individually effective in improving patients with CNSNP. However, from the results obtained questions remain requiring new randomised studies to find the answers, i.e. whether the same results can be obtained as a function of the participant’s age or the optimal frequency (number of weeks) to achieve the best cost-effectiveness balance.

## Conclusion

After 8 weeks of a MPP that integrated land-based physical therapeutic exercises, health education and adapted swimming, clinically relevant improvements in disability, physical and mental health states and quality of life were observed in patients who suffer CNSNP. The clinical efficacy requires verification using a randomised controlled study design.

## Supporting Information

S1 TREND Checklist (PDF)

S1 Protocol (DOC)
