# Supplementary material for: Changes in Disability, Physical/Mental Health States and Quality of Life during an 8-Week Multimodal Physiotherapy Programme in Patients with Chronic Non-Specific Neck Pain: A Prospective Cohort Study
Source: PLoS One. 2015 Feb 24;10(2):e0118395. doi: 10.1371/journal.pone.0118395 (PMC4339981; doi:10.1371/journal.pone.0118395)
Supplement: S1 Protocol — (DOC) [file pone.0118395.s002.doc]

**CUESTIONARIO PARA EVALUACIÓN POR EL COMITÉ**

**ÉTICO DE EXPERIMENTACIÓN**

| **DATOS PERSONALES** | | |
| --- | --- | --- |
| Nombre investigador principal: | | Antonio I. Cuesta Vargas |
| Otros investigadores: | | Manuel Gonzalez Sanchez |
| Dirección de correspondencia: | | Univesidad de Málaga  Facultad de Ciencias de la Salud.  Paseo de Martiricos sn. 29071  Málaga (España) |
| Teléfono: | | 952 137551  952 137071 |
| Email | | acuesta@uma.es  mgsa23@uma.es |
|  | |  |
| **Datos del estudio** | | |
| Titulo del estudio | Fisioterapia multimodal como intervención en pacientes con cervicalgia crónica inespecífica crónica. | |
| Fecha de desarrollo | Nov 2011 – Oct 2012 | |
| Duracion: | 1 año | |
| **Objetivo del estudio** | | |
| El objetivo de este estudio es analizar el efecto de un programa multimodal de fisioterapia, donde se integran el ejercicio físico terapéutico, la educación sanitaria y la natación adaptada como medio para intervenir en pacientes que sufren cervicalgia crónica inespecífica. | | |
| **PARTICIPANTES**  Se prentende reclutar a usuarios de una instalación de deportiva comunitaria (Patronato Municipal de Deportes de Torremolinos) en base a los criterios de inclusión / exclusión establecidos (y expuestos a continuación), para que participen en este estudio prospectivo.   - Criterios de inclusión: - Edad comprendida entre 18 y 65 años. - Sufrir cervicalgia crónica inespecífica con una duración o persistencia igual o superior a 12 semanas. - Criterios de exclusión: - Cervicalgia debido a una causa identificable: Hernia de disco, fractura espinal, metástasis cancerosa, escoliosis severa o progresiva, infección, estenosis espinal, pérdida de peso inesperada, espondilolistesis, osteoporosis, inflamación reumática. - Cirugía en la columna vertebral en los últimos doce meses. - Accidente de tráfico en los últimos 3 meses. - Radiculopatía cervical. - Embarazo. - Pacientes que puedan sufrir alguna condición que pudieran afectar a los resultados: depresión, tratamiento médico para la cervicalgia, afecciones en corazón o hígado, fibromialgia severa, medicación para el tratamiento del SIDA o la hepatitis. - Personas que no sepan nadar. - Personas con dificultad en el tratamiento: demencia, graves problemas adictivos, parálisis, visión deficitaria. - NO Entregar el consentimiento informado firmado. | | |
| **VARIABLES DE RESULTADO**  Las variables de resultado serán:   - Discapacidad: Medida con la versión española del cuestionario Neck Disability Index (NDI). Esta herramienta presenta una fiabilidad de 0.978. - Estado general de salud física y mental: Medido mediante el uso de la versión española del cuestionario SF-12. Esta herramienta presenta una fiabilidad que oscila entre 0.86 y 0.89. - Calidad de vida: Medida con la versión española del cuestionario EuroQoL-5 Dimensiones. Esta herramienta presenta una fiabilidad que oscila entre 0.86 y 0.90.   **INTERVENCIÓN**  La intervención en este estudio consistirá en aplicar un programa multimodal de fisioterapia que integrará ejercicio físico terapéutico, educación sanitaria y natación adaptada durante 8 semanas.  Cada sesión estará dividida en dos partes de 30 minutos. En la primera parte se desarrollarán ejercicios físicos terapéuticos en seco dedicados a la mejora de la movilidad, el control motor, la fuerza y la resistencia de los músculos del cuello.  La mejora de la movilidad se realizará mediante el auto-estiramiento de las barreras miotendinosas del pectoral, angular de la escápula, parte alta del trapecio, escalenos y esternocleidomastoideo. El control motor se mejoró mediante la activación del sistema local durante 10 segundos, manteniendo la columna vertebral en posición neutra (valorando la columna vertebral completa, desde la pelvis hasta la cabeza) durante actividades el desarrollo de actividades de la vida diaria (sentarse, levantarse, subir escaleras, utilizar el ordenador,…), utilizando, como feedback, un espejo y el comentario del fisioterapeuta que | | |
| supervisaba las sesiones.  La resistencia de los músculos profundo del cuello se pretende mejorar mediante el uso de ejercicios de sinergia de brazos (poleas, presas…). De cada ejercicio se realizarán tres series de 15 repeticiones cada una. El tiempo de cada serie será de 30 segundos con otros 30 segundos de descanso entre ellas, donde la columna cervical permanecerá en posición neutra durante la ejecución. En el protocolo de ejercicios terapéuticos, también se incluirán  En el protocolo de ejercicios terapéuticos, y con el mismo criterio, también se incluirán ejercicios con bandas elásticas para activar los fijadores de la escápula, incluyendo ejercicios de tracción y de empuje.  La segunda parte de la sesión consistirán en 30 minutos de natación adaptada con ejercicios aeróbicos, manteniendo el cuello en posición neutra. Un tubo para respirar permitirá la ejecución de los ejercicios sin sobrecargar las estructuras del cuello. La natación ventral con el tubo reducirá el peso percibido de la cabeza debido a la flotación y evitará cualquier movimiento del cuello para respirar.  La educación sanitaria será propuesta mediante un decálogo de cervicalgia inespecífica crónica así como mediante el uso una constante repetición de consejos breves que el fisioterapeuta proporcionará durante la supervisión de los ejercicios.  Todos los participantes serán animados para que su participación en el programa multimodal de fisioterapia sea activa, enfatizando la importancia de la adherencia para mejorar los resultados de la intervención.  El fisioterapeuta que supervisará la sesión está cegado en cuanto al conocimiento de qué participantes formarán parte del estudio. Todo aquel participante cuya adherencia al tratamiento sea inferior al 90%, será excluido del análisis estadístico de los mismos. | | |
| **SALUD Y SEGURIDAD** | | |
| La seguridad de este estudio está garantizada en todo momento debido al hecho de que han sido excluidos del mismo aquellas personas que no sean capaces de realizar los ejercicios propuestos o que no sean capaces de nadar.  Además, en la segunda parte de las sesiones, donde se desarrollará el protocolo de natación adaptada, cada participante llevará un flotador en la cintura que facilitará la ejecución de los movimientos y garantizará su seguridad. Además, durante todo el desarrollo de las sesiones, un fisioterapeuta supervisará el correcto desarrollo de las actividades. Para garantizar este hecho, el número de personas que formarán parte de cada grupo de intervención, oscilará entre 8 – 10 sujetos. | | |
| **PUBLICACIÓN DE RESULTADOS** | | |
| A los resultados de este estudio se le pretende dar la mayor difusión posible procurando su publicación en las mejores revistas internacionales cuyas exigencias de publicación sean, entre otras, la revisión por pares del los documentos. | | |
|  | | |
| **ALMACENAMIENTO DE DATOS** | | |
| Los datos serán almacenados de manera codificada en un ordenador protegido por clave de acceso y solo los dos investigadores de este proyecto (principal y colaborador) tendrán acceso a los mismos.  Los datos personales serán almacenados separadamente, en un ordenador con clave de acceso, pudiendo accede a ellos solo el investigador principal.  Así, se pretende garantizar en todo momento el anonimato de cada uno de los participantes así como la perfecta custodia de datos personales. | | |

**CUESTIONARIOS**

**Cuestionario A**

**¿Se trata de investigaciones que implique intervenciones en humanos, incluyendo toma de muestras biológicas?**

| - SI | - NO |
| --- | --- |

En caso afirmativo

1. ¿Se garantiza que la participación de los sujetos experimentales en la práctica es voluntaria y libre?

| - SI | - NO |
| --- | --- |

1. ¿Se garantiza la confidencialidad de los datos?

| - SI | - NO |
| --- | --- |

1. En el consentimiento informado:

- ¿Se hace explícito el compromiso de confidencialidad ante los sujetos que participan en el estudio?

| - SI | - NO |
| --- | --- |

- ¿Se informa adecuadamente a los sujetos del procedimiento y los riesgos o molestias que pudieran derivarse de la práctica y la forma en que son controlados o mitigados?

| - SI | - NO |
| --- | --- |

- ¿Se informa de las medidas para asegurar una compensación adecuada si sufre algún daño o molestia causada por la investigación?

| - SI | - NO |
| --- | --- |

- ¿Se informa de la posibilidad de ser informado, si así lo desea, de los datos que se obtengan durante la investigación y la forma de obtener dicha información?

| - SI | - NO |
| --- | --- |

(El CEUMA podrá en todo mometo solicitar información sobre el consentimiento informado y la hoja de información a los sujetos)

1. ¿Participan personas especialmente vulnerables, como menores de edad, ancianos, incapacitados, personas con trastornos mentales, etc.?.

| - SI | - NO |
| --- | --- |

1. Indique si existen posibles riesgos o molestias que pudieran derivarse de la práctica y la forma en que son controlados o mitigados.

| - SI | - NO |
| --- | --- |

En caso afirmativo explique la forma en que son controlados o mitigados: (máximo 500 caracteres)

*La seguridad de este estudio está garantizada en todo momento debido al hecho de que han sido excluidos del mismo aquellas personas que no sean capaces de realizar los ejercicios propuestos o que no sean capaces de nadar.*

*Además, en la segunda parte de las sesiones, donde se desarrollará el protocolo de natación adaptada, cada participante llevará un flotador en la cintura que facilitará la ejecución de los movimientos y garantizará su seguridad. Además, durante todo el desarrollo de las sesiones, un fisioterapeuta supervisará el correcto desarrollo de las actividades. Para garantizar este hecho, el número de personas que formarán parte de cada grupo de intervención, oscilará entre 8 – 10 sujetos.*

1. ¿Cuál es el método de disociación de datos?:

- Codificación (Disociación reversible).

| - SI | - NO |
| --- | --- |

- Anonimización (Disociación irreversible)

| - SI | - NO |
| --- | --- |

1. ¿Se realizan pruebas de tipo genético?

| - SI | - NO |
| --- | --- |

- En caso afirmativo indicar si se cuenta con una persona responsable de informar sobre los resultados al paciente (máximo 100 caracteres)

**Cuestionario B.**

1. ¿Se trata de investigaciones que implique Investigación con muestras biológicas almacenadas?

| - SI | - NO |
| --- | --- |

En caso afirmativo

- ¿Se necesita nuevo consentimiento para esta investigación?

| - SI | - NO |
| --- | --- |

1. ¿Cual es el método de disociación de datos?

- Codificación (Disociación reversible).

| - SI | - NO |
| --- | --- |

- Anonimización (Disociación irreversible)

| - SI | - NO |
| --- | --- |

**Cuestionario C.**

1. ¿Se trata de investigaciones que implique utilización de datos de origen humano?

| - SI | - NO |
| --- | --- |

En caso afirmativo

1. ¿Se necesita nuevo consentimiento para esta investigación?

| - SI | - NO |
| --- | --- |

1. Cual es el método de disociación de datos:

- Codificación (Disociación reversible).

| - SI | - NO |
| --- | --- |

- Anonimización (Disociación irreversible)

| - SI | - NO |
| --- | --- |

**EN TODOS LOS CASOS**:

1. ¿Se ofrecen incentivos o compensaciones a los sujetos por su participación en los experimentos?

| - SI | - NO |
| --- | --- |

- En caso afirmativo indique su naturaleza y cuantía. (máximo 500 caracteres)

1. ¿Se ofrecen incentivos o compensaciones a los investigadores?

| - SI | - NO |
| --- | --- |

- En caso afirmativo indique su naturaleza y cuantía. (máximo 500 caracteres)

1. ¿Existe algún tipo de contraprestación y/o seguro para los participantes que cubran daños en caso de producirse?.

| - SI | - NO |
| --- | --- |

- En caso afirmativo indique su naturaleza y cuantía. (máximo 500 caracteres)

1. El estudio se realizará en las instalaciones idóneas para llevar a cabo los diseños experimentales.

| - SI | - NO |
| --- | --- |

COMPROMISO ESCRITO DEL INVESTIGADOR

RESPONSABLE DEL PROYECTO

**Título del Proyecto**: Fisioterapia multimodal como intervención en pacientes con cervicalgia crónica inespecífica crónica.

**Investigador** **Responsable**: Antonio I. Cuesta Vargas.

**Departamento**: Psiquiatría y fisioterapia.

El investigador principal que firma la propuesta declara ser conocedores de la idoneidad de los procedimientos y protocolos que van a realizar relativos a la experimentación con humanos. Declaran tanto el investigador como el equipo de investigación

- Han tenido en cuenta la información suficiente que asegure la inocuidad o el beneficio para las personas que participan en la experimentación y ha considerado métodos alternativos a la experimentación con humanos que permita de alcanzar conclusiones válidas?
- Se garantiza la trazabilidad de tejidos y cualquier material biológico de origen humano, para asegurar las normas de calidad y seguridad.
- Han tomado medidas encaminadas a proteger la vida, la salud, la dignidad, la integridad, el derecho a la autodeterminación, la intimidad y la confidencialidad de la información personal de las personas que participan en investigación
- Las acciones sobre las personas que participan en la experimentación están supervisadas por un médico u otro profesional de la salud competente y cualificado apropiadamente. Igualmente, el personal que participa en los procedimientos posee la titulación y tiene la formación adecuada para llevar a cabo las tareas que se les encomienda
- En su caso, se han tomado medidas encaminadas a no dañar el medio ambiente y se procura el bienestar de los sujetos que participan de la investigación. El investigador abajo firmante declara conocer y atenerse a lo dispuesto en la normativa legal vigente. Igualmente, asume que el informe del comité de ética de la Universidad de Málaga se referirá únicamente al procedimiento recogido en este impreso, por lo que cualquier modificación, sin solicitar una nueva aprobación por parte del CEUMA, será responsabilidad exclusiva del solicitante.
